# Supplementary material for: Genetic analysis of F1 cluster phages that infect Mycobacterium smegmatis identifies two distinct holin-like proteins that regulate the host lysis event
Source: PLoS One. 2026 Apr 7;21(4):e0339202. doi: 10.1371/journal.pone.0339202 (PMC13056207; doi:10.1371/journal.pone.0339202)
Supplement: S2 File — (PDF) [file pone.0339202.s002.pdf]

S2 Figure 1. One-step growth curves schematic protocol

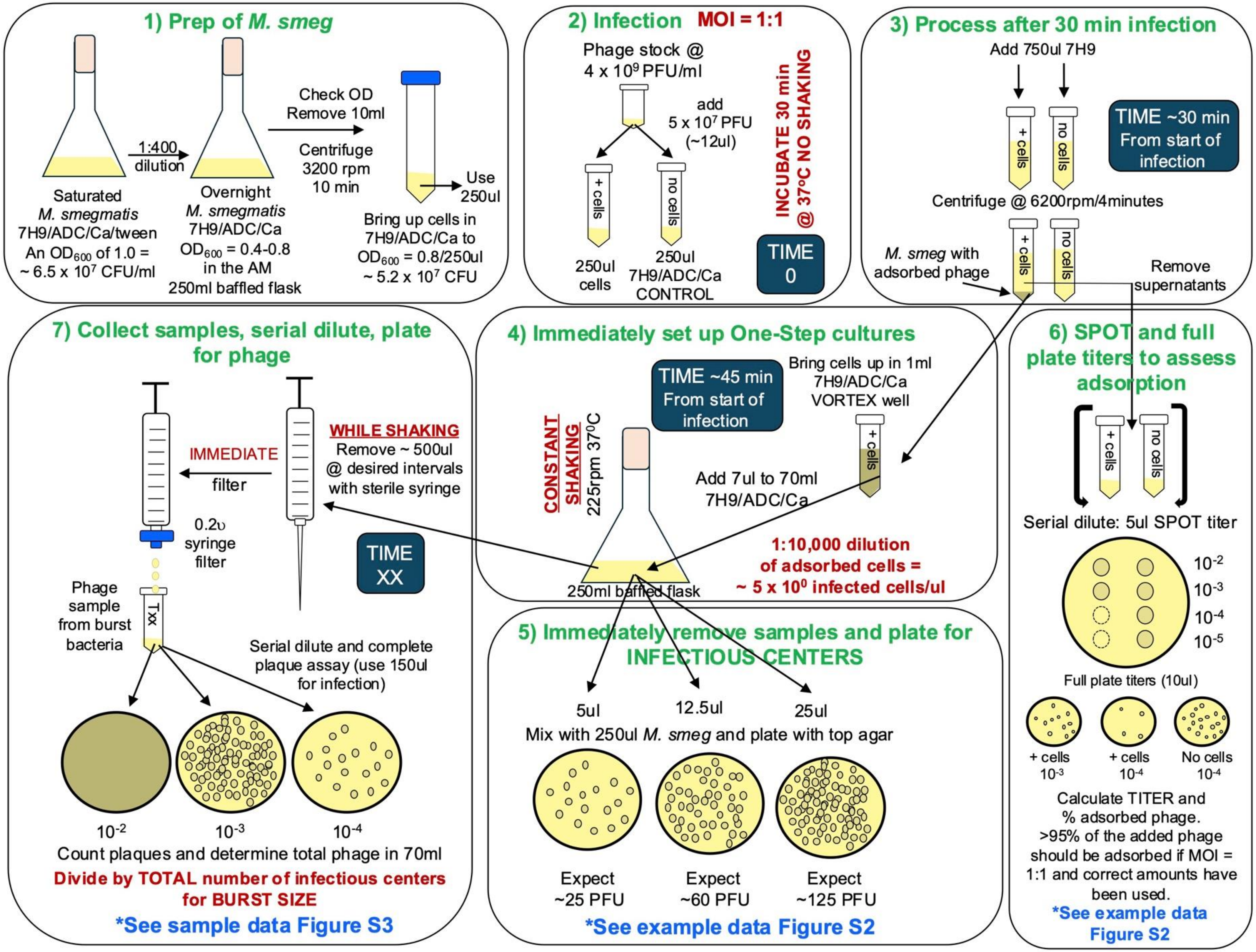

S2 Figure 1. One-step growth curves schematic protocol. The schematic of the One-Step growth curves procedure described in Methods. See example Titer and Infectious Centers example data set in S3 Figure 2. See example Burst Size data set in S4 Figure 3.

S3 Figure 2. One-step example data set: titers, adsorption and infectious centers

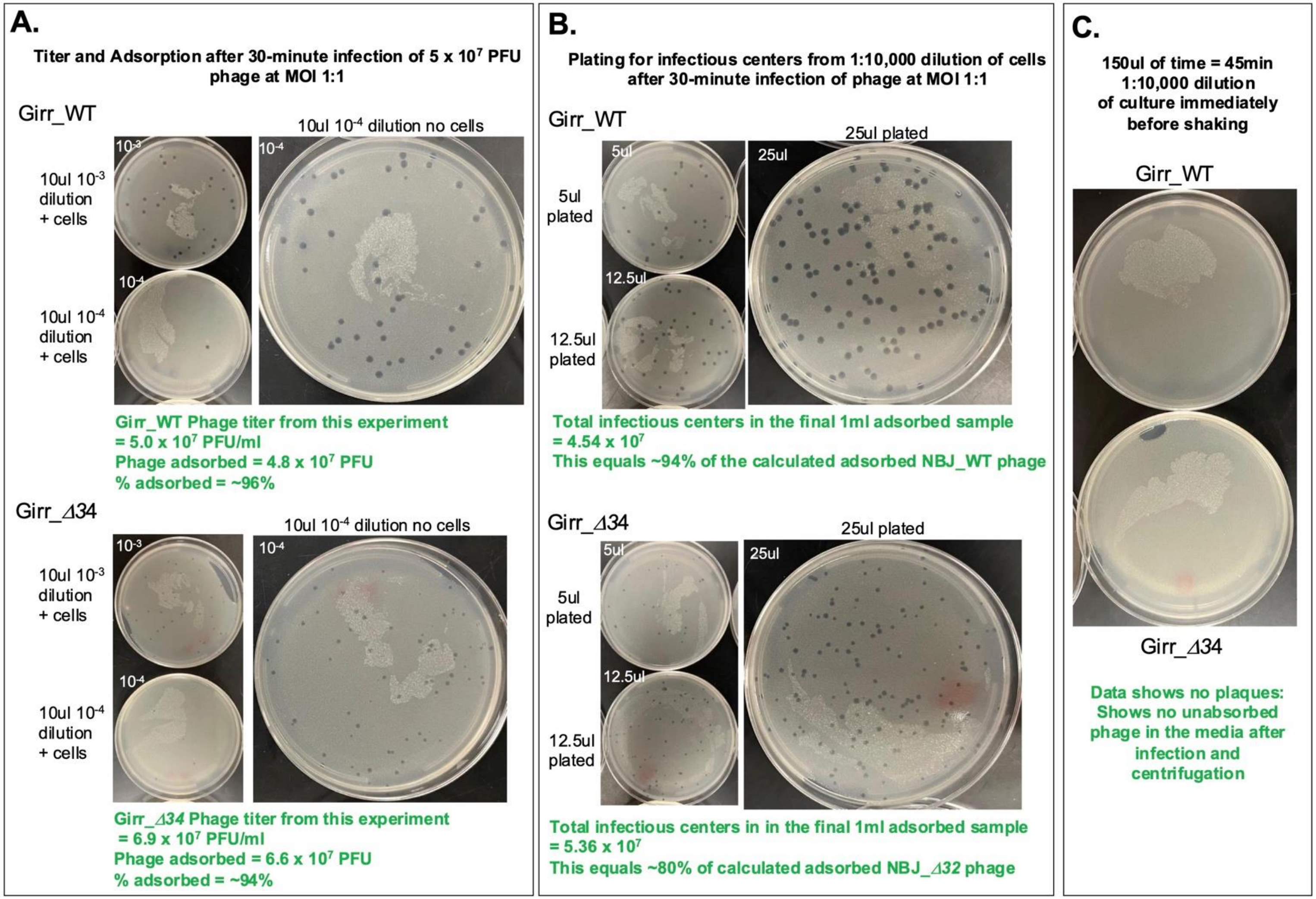

S3 Figure 2. One-step example data set: titers, adsorption and infectious centers. **A.** Girr_WT and Girr_*Δ34* titer and adsorption analysis. 5 x 10^7 PFU of indicated phage was mixed with 5 x 10^7 *M. smegmatis* cells in 250ul 7H9 media or 250ul 7H9 media with no cells. Samples were incubated for 30 min., added to 750ul 7H9 media, centrifuged and the supernatant serial diluted as shown and used for plaque assay. Resulting plates, titers and phage adsorption is shown. **B.** The cell pellet from A, was brought up in 1ml of 7H9 media, vortexed and 7ul added to 70ml 7H9 media. 5ul, 12.5ul and 25ul was removed for plaque assay and resulting plates are shown. Number of plaques equal infectious centers @ T_{45}min of the experiment. **C.** Free phage analysis. 150ul of the culture described in B was used for plaque assay @ T_{45}min and the resulting plates are shown. No plaques are detected indicating that the unadsorbed free phage in the samples is minimal and the data in B represents plaques formed by infected cells and not residual free (unabsorbed) phage.

S4 Figure 3. One-step example data set: Girr_WT

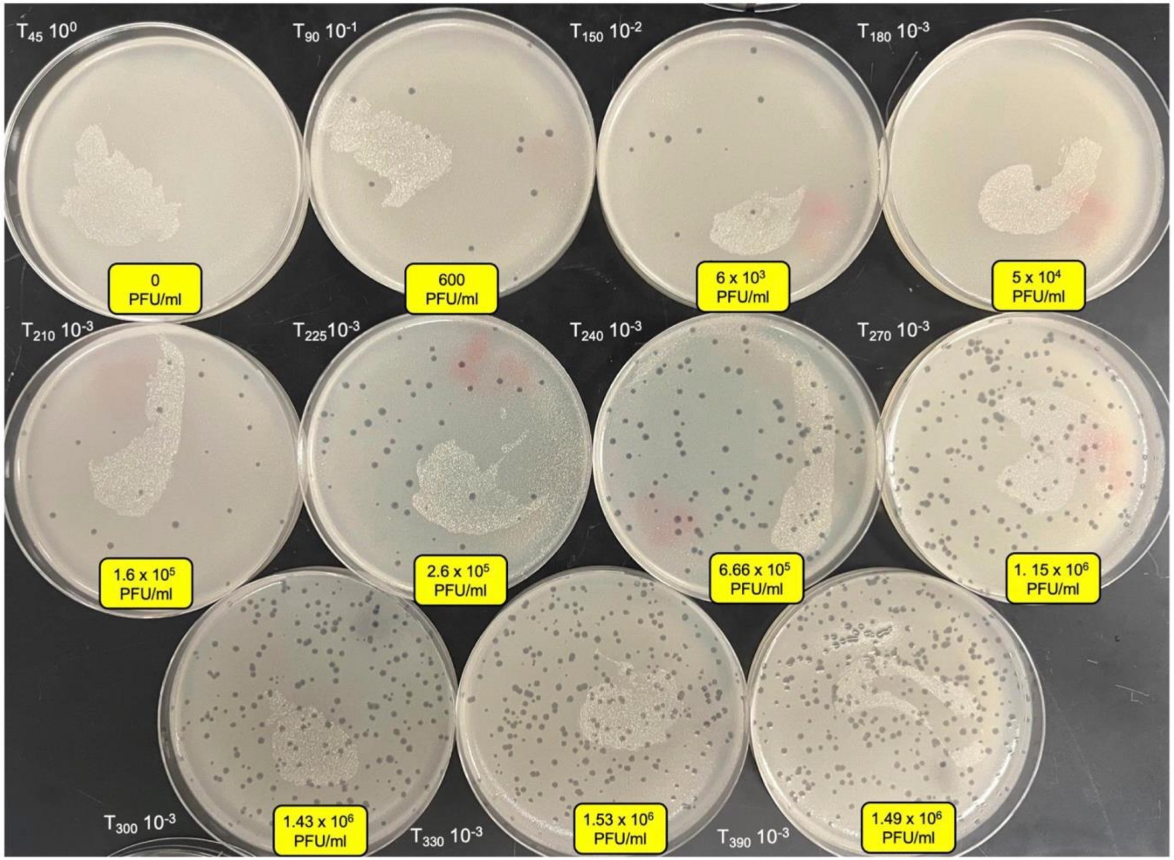

S4 Figure 3. One-step example data set: Girr_WT. The Girr_WT sample described in Figure S2 was harvested at the indicated times and immediately passed through a 0.2μ filter to remove bacterial cells as described in Methods. Samples were serial diluted and 150μl of each sample used for plaque assay. A representative plate from one of the dilutions at each time point is presented. The titer at each time point was determined by counting all the plaques, dividing by 150μl and then multiplying by 1000 to obtain the titer in PFU/ml. Note that the titer plateaus at 300 minutes. In this representative experiment the maximal burst size would be at T_{330} and determined by the formula: 1.53×10^6 PFU/ml (titer @ T_{330}) x 70ml/3.18×10^5 infectious centers (total infectious centers in the 70ml culture) = 336 PFU/cell. The average burst across the plateau of the experiment would include the bursts at T300 and T390, or 314 + 327 + 336 = 325.7 PFU/cell.

S5 Figure 4. AlphaFold3 predictions of Girr and NBJ Lysin A

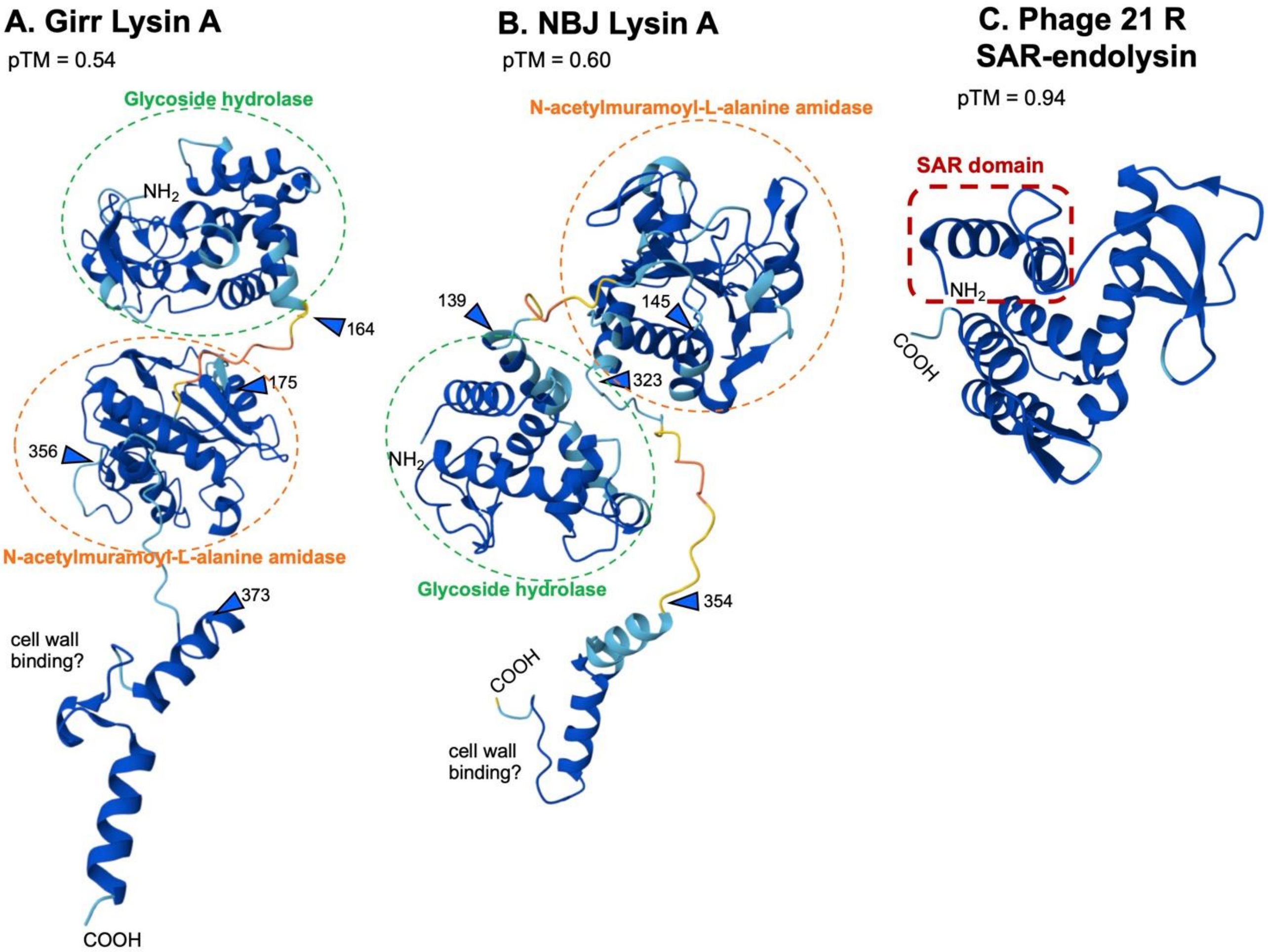

S5 Figure 4. AlphaFold3 predictions of Girr and NBJ Lysin A. All protein sequences were evaluated using AlphaFold3 as described in Methods. N and C-terminal domains are identified by HH_2 and COOH. Numbers indicate amino acid location. **A.** Girr Lysin A. The two catalytic domains predicted by HHpred are shown and the extended C-terminal domain thought to be involved in cell wall binding [21]. **B.** NBJ Lysin A. The two catalytic domains predicted by HHpred are shown and the extended C-terminal domain thought to be involved in cell wall binding [21]. **C.** Protein R (SAR-endolysin) from phage 21. The SAR domain is identified with hatched box [13, 14].

S6 Figure 5. AlphaFold3 predictions of Girr LysF1a and phage 21 $S^{21}68$

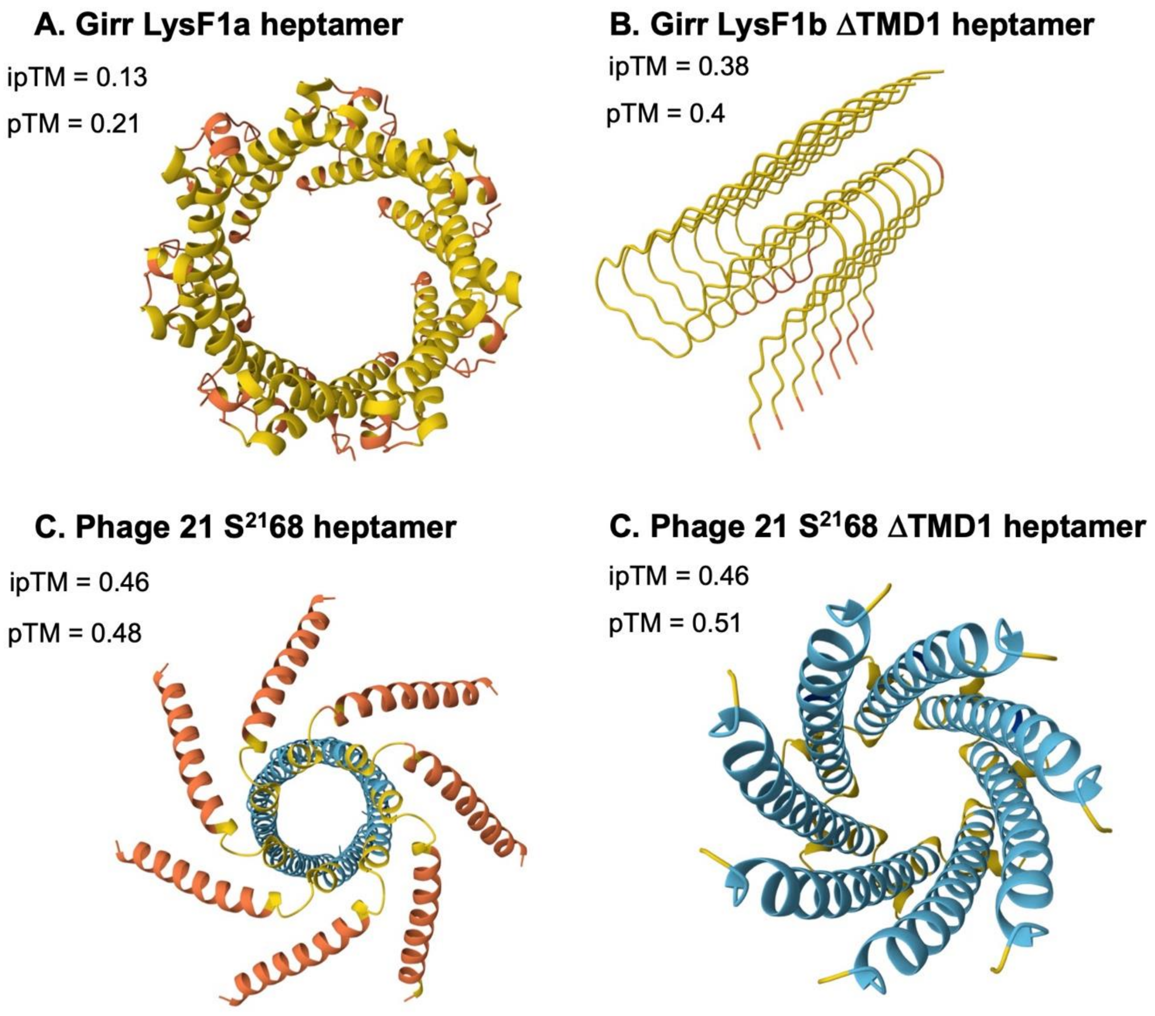

S6 Figure 5. AlphaFold3 predictions of Girr LysF1a and phage 21 $S^{21}68$. All protein sequences were evaluated using AlphaFold3 as described in Methods. It is proposed that the TMD2 of Phage 21 $S^{21}68$ forms the main lesion as a heptamer and that the TMD1 is extracellular and dispensable for hole formation [4, 76, 77]. **A.** Prediction of full-length Girr LysF1a as heptamer. **B.** Prediction of Girr LysF1a without TMD1 as heptamer. **C**. Prediction of full-length Phage 21 $S^{21}68$ pinholin as heptamer. **D.** Prediction of Phage 21 $S^{21}68$ without TMD1 heptamer.

S7 Figure 6. Deletion of NBJ *lysF1a* and *lysF1b*

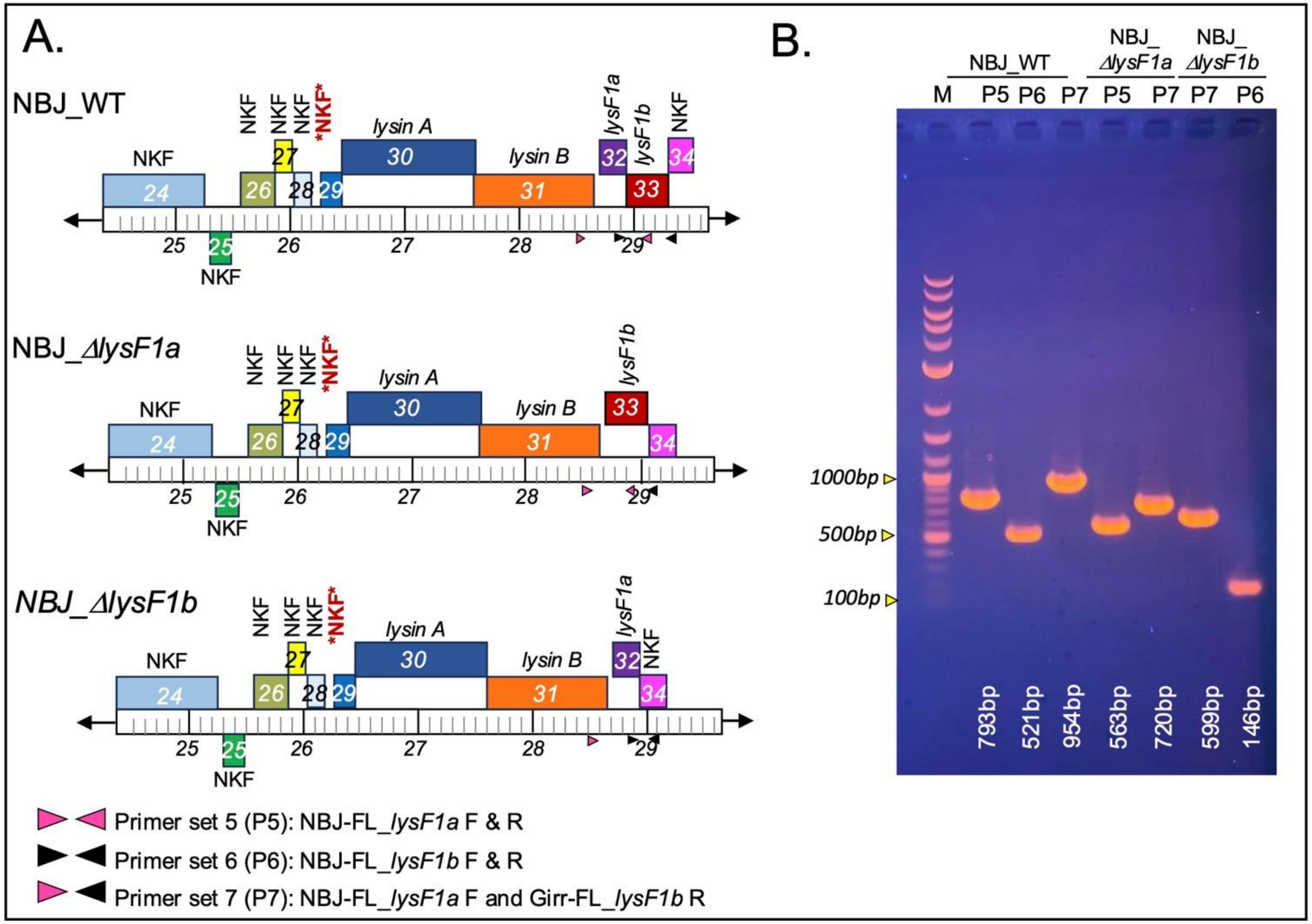

S7 Figure 6. Deletion of NBJ *lysF1a* and *lysF1b*. A. Genomic structure and PCR primer set locations in NBJ gene deletion mutants. Primer set 1 (P5) uses primers NBJ-FL_*lysF1a* (F and R) and generates a 793bp fragment in NBJ_WT and 536bp with *lysF1a* deletion. Primer set 6 (P6) uses primers NBJ-FL_*lysF1b* (F and R) and generates a 521bp fragment in NBJ_WT and 146bp with *lysF1b* deletion. Primer set 7 (P7) uses the forward NBJ-FL_*lysF1a* and reverse NBJ-FL_*lysF1b* and generates a 954bp fragment in NBJ_WT and either 720bp with *lysF1a* deletion or 599bp with *lysF1b* deletion. **B.** PCR verification of high titer lysates of NBJ_WT, NBJ_Δ*lysF1a* and NBJ_Δ*lysF1b*. PCR band sizes are identified and verify the deletion of the associated gene *32* or *33* in each mutant NBJ phage.

S8 Figure 7. Infection and adsorption of Girr_WT and TMD mutants

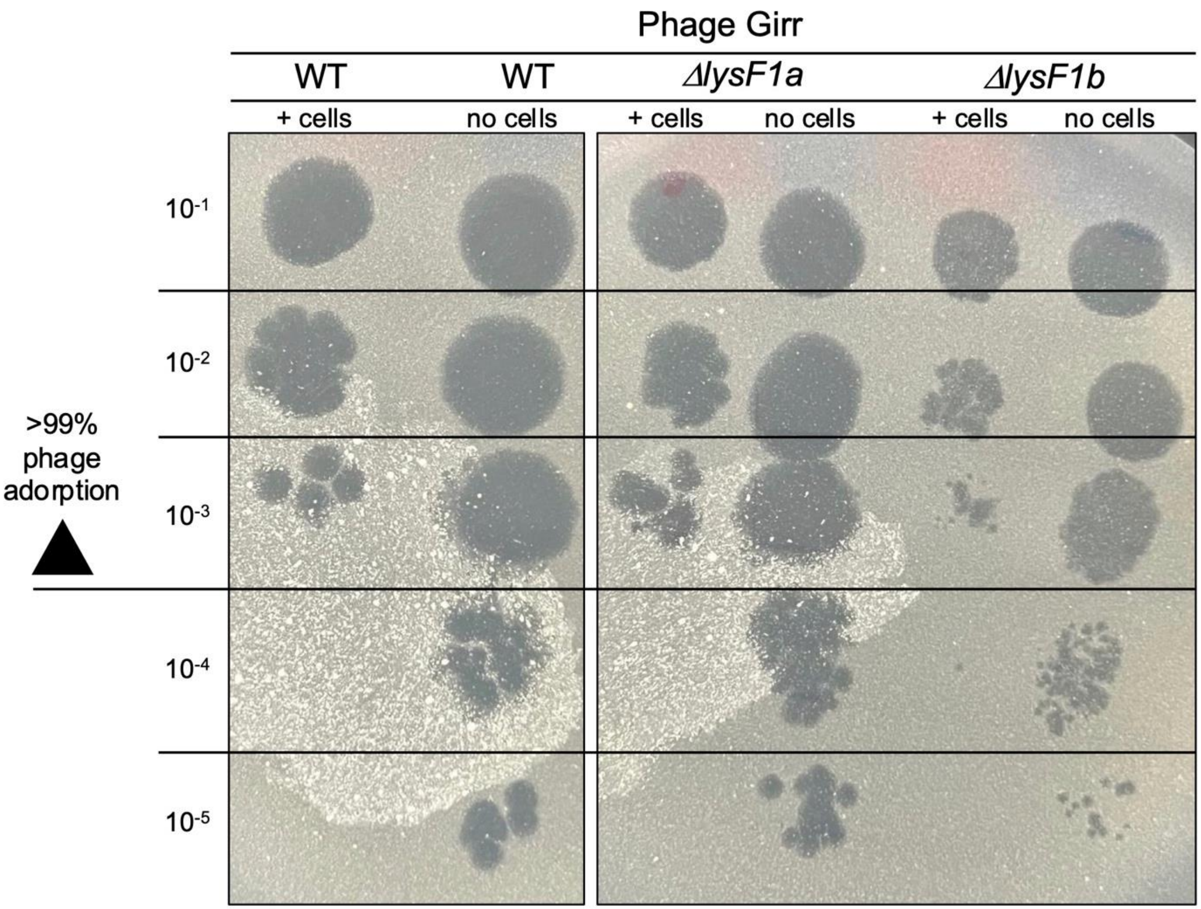

S8 Figure 7. Infection and adsorption of Girr_WT and TMD mutants. 5 x 10^7 PFU of indicated phage was mixed with 5 x 10^7 *M. smegmatis* cells in 250ul 7H9 media or 250ul 7H9 media with no cells. Samples were incubated for 30 min., added to 750ul 7H9 media, centrifuged and the supernatant serial diluted as shown and 5ul spotted onto a lawn of *M. smegmatis.* Girr_WT, Girr_Δ*LysF1a* and Girr_Δ*lysF1b* all showed >98% adsorption in this assay and also the same efficiency of plating.

S9 Figure 8. Plaque sizes in NBJ_WT, NBJ_Δ*lysF1a* and NBJ_Δ*lysF1b*

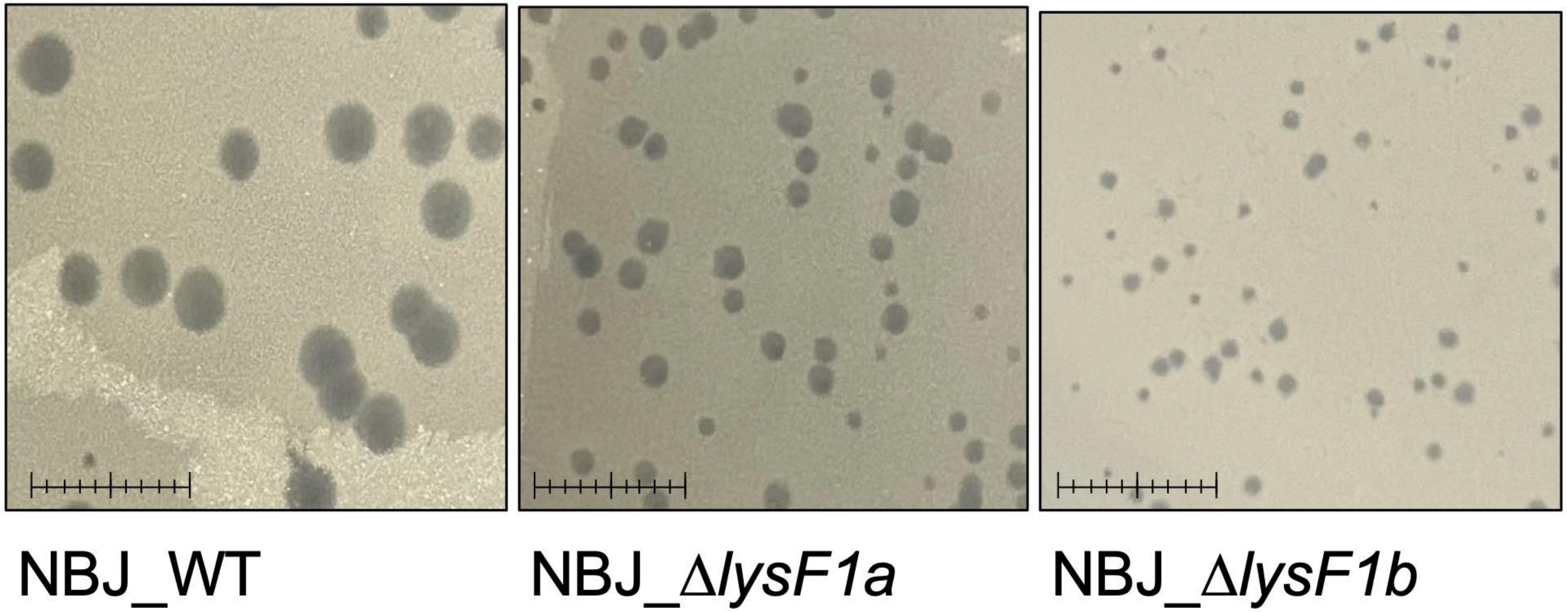

S9 Figure 8 Plaque sizes in NBJ_WT, NBJ_Δ*lysF1a* and NBJ_Δ*lysF1b*. *M. smegmatis* was infected with identical numbers of phage and plaque assay performed as described in Methods. All plates were incubated for 36 hrs. at 37°C. Scale = 1cm . Scale = 1cm.

S10 Figure 9. Genomic context of lysis cassette genes in Girr and NBJ

A. Girr genes *33, 34, 35*

ACGACGCGGCCGGGAAGAACCAGACCGCGATCGCCCGGATCATCACGGAGAACTCGTGGATCGGCGGCCCGACCGCGTTG
TTCTCGCGGGTGCTCACACTGTTCGGAAACCCGGTCGGTGAGGGCTTCGGCATCGTCAAGGCGATCTTCGACGCCGTCAT
GTTCCTCGCCGCCAACCCCAATCCGCACTACTCGACGTTCGCCACACCGGGCGACGTGGAGTGGATGCGCGGCGTCGCGA
CC**TGACCCCGCGCTGTCCGACAACCCAACCATCAGAGGGGAAATG**CAAGCCATGTTGACACGTTCGTTTTGGATCGACGC
CGCCGAACGCGCGGCCCGCACGTTCGCCCAAACCGCGATCGCCACACTCGGCGCGGGCGCGGTTGACCTACTCGCCACCG
ATTGGGTGTCAGTGCTGTCAGTGTCCGGCGGCGCCGCAGTGGTGTCACTGCTGATGTCTATCGGCGCGGAACGCCGCGGC
AACCCCGGAACGGCTTCGGCGACTAGAGCGGTCACTGCCGC**ATGA**TCTGGGAATCGGTGCGCGAAGCGGTGGACGCCGCG
TACCAGCCAGACGACGGTATCGACCTGATAGGACTGCTCATCATCGGTTTACCTTCCACGATCGCAGCTATCGGAACGGG
AATTGTCGGTGTCCTCACTGTTCGAGGGCAACGCAAGGCCGGGAACGTGCCAGACGGATCGACGCGAAAACCTATGAGA
TTCACGAGCAGACCGTCAACACCCATGACACCAACATGCGCGACGACCTCGACGAGATACGCGATCTGGTGCGGGACGGA
TTCAAACAGATTCAACGGGACATCGGAGGGTTGAGGGAGGAACTGCGAACCGAACGCCTCGAACGCATCGAAGGCGACAA
GCGACGCGACCGG**TGA**

B. NBJ genes *31, 32, 33*

ATGGCCCCATCGAACACTCAGGGCGTCTCATCGAACGGTATGCGTGACACCCCGCCGTGGTGGCGCGACTACGCACACC
AGGGCGACTTGTACGCGTGCACCGAACCGGGCGACACACAAGAGGTCCGCAACGCCATCTGGCAGATCGTGCGCGACCT
GGACCTGTTCACCGGACCCGATTCGCTACTCGCCCAAGTAATCGAACTTGTGCAGGCCCCGCTACCGGAGACGATCGCG
ATCACCAAAGCGATCCTCGACGCCGGCATGTTCTTCGCGAAACGCACCGGCCCGCACGTGGACTACAACCCCCAGCCCG
CCATCGACTACCTACGCACA**TAGGAGGCACCATG**CTGACACGTTCATTCTGGATCGACGCCGCCGAGCGGGCCATACGC
ACATTCGCCCAAACCGCGATCGCCACACTCGGCGCCGGGGCAGTCGACCTGATGACCACCGACTGGATATCGGTGCTGT
CCGTGTCCGGCGGCGCGGCCGTCGTATCACTGCTGATGTCTATCGGCGCCGAACGCCGCGGCAACCCCGGAACGGCGTC
GGCCACTAGAGCGGTCACCGCCGCA**TGA**TCTGGGAATCGGTGCGCGAAGCAATGGATGCCGCGTACCAGCCCGAAGATG
GTATCGACCTGATAGGACTGCTCATCATCGGTTTACCTTCCACGATCGCAGCGATCGGAACGGGAATTGTCGGTGTCCT
CACTGTTCGAGGGCAACGCAAGGCCGGGAACGTGCCCGACAGATCGACGCGAAAACCGATGAGATTCACGAGCAGACC
GTCAACACCCATGACACCAACATGCGCGACGACCTCGACGAGATACGCGATTTGGTGCGCGACGGCTTCAAACAGATTC
AACGGGACATCGGAGGGTTGAGGGAGGAGCTGCGAACCGAACGCCTCGAACGCATCGAAGGCGACAAGCGACGCGACCG
G**TGA**

S10 Figure 9. Genomic context of lysis cassette genes in Girr and NBJ. A. Sequence of Girr genes *33, 34* and *35*. Black font: The terminal portion of the *lysin B* gene 33. Yellow highlighted region: the intergenic region between gene *33* and gene *34*. Blue font: *lysF1a* gene *34*. Brown font: *lysF1b* gene *35*. Start codons are highlighted in green and stop codons are red font. The ATGA 4bp overlap of genes 34 and 35 are shown. **B.** Sequence of NBJ genes *31, 32* and *33*. Black font: The terminal portion of the *lysin B* gene 31. Yellow highlighted region: the intergenic region between gene *31* and gene *32*. Blue font: *lysF1a* gene *32*. Brown font: *lysF1b* gene *33*. Start codons are highlighted in green and stop codons are red font. The ATGA 4bp overlap of genes 34 and 35 are shown.

S11 Figure 10. Energy poisons and $CHCl_3$ treatment trigger early lysis by Girr_WT and NBJ_WT

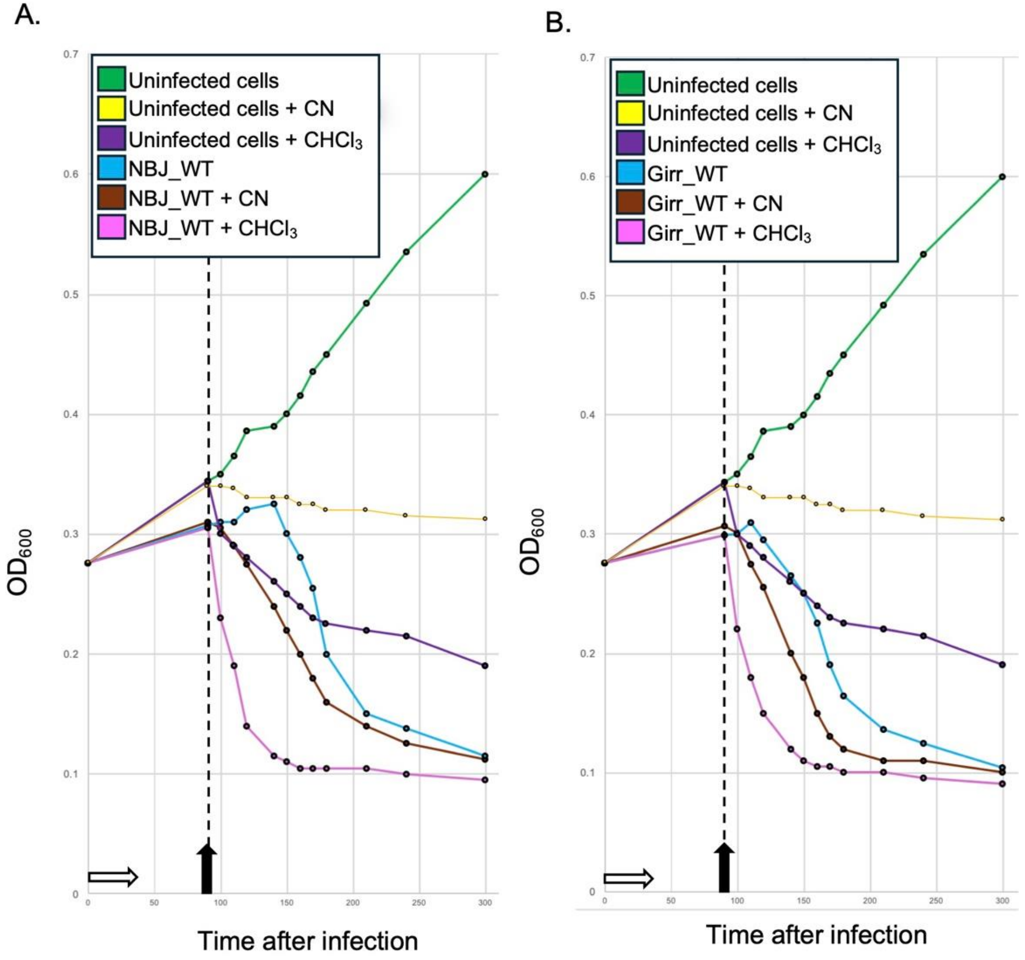

S11 Figure 10. Energy poisons trigger early lysis in phage expressing LysF1b but not LysF1a. At T_0 a log growth culture of *M. smegmatis* at an OD_{600} of ~0.25 was infected with the indicated phage at an MOI of 10:1 and incubated at 37°C for 30 minutes without shaking (open arrow). Control cultures (no phage) did not receive phage. At T_{30} minutes the culture was constantly shaken a 225rpm, aliquots removed at the indicated times and bacterial growth evaluated at OD_{600}. At T = 90-minutes (black arrow and dashed line), cells were treated with KCN (final concentration 10mm) or $CHCL_3$ (final concentration 1%). **A**. Representative results following infection with NBJ_WT. At T = 90-minutes (black arrow and dashed line), cells were treated with CN (final concentration 10mm) or $CHCL_3$ (final concentration 1%). **B**. Representative results following infection with Girr_WT. At T = 90-minutes (black arrow and dashed line), cells were treated with CN (final concentration 10mm) or $CHCL_3$ (final concentration 1%). NOTE: the results in A and B were completed simultaneously, thus, the uninfected cells serves as the control for both data sets. The Girr and NBJ data is plotted on separate graphs for ease of review.

S12 Figure 11. Cell viability of *M. smegmatis* infected with NBJ phages

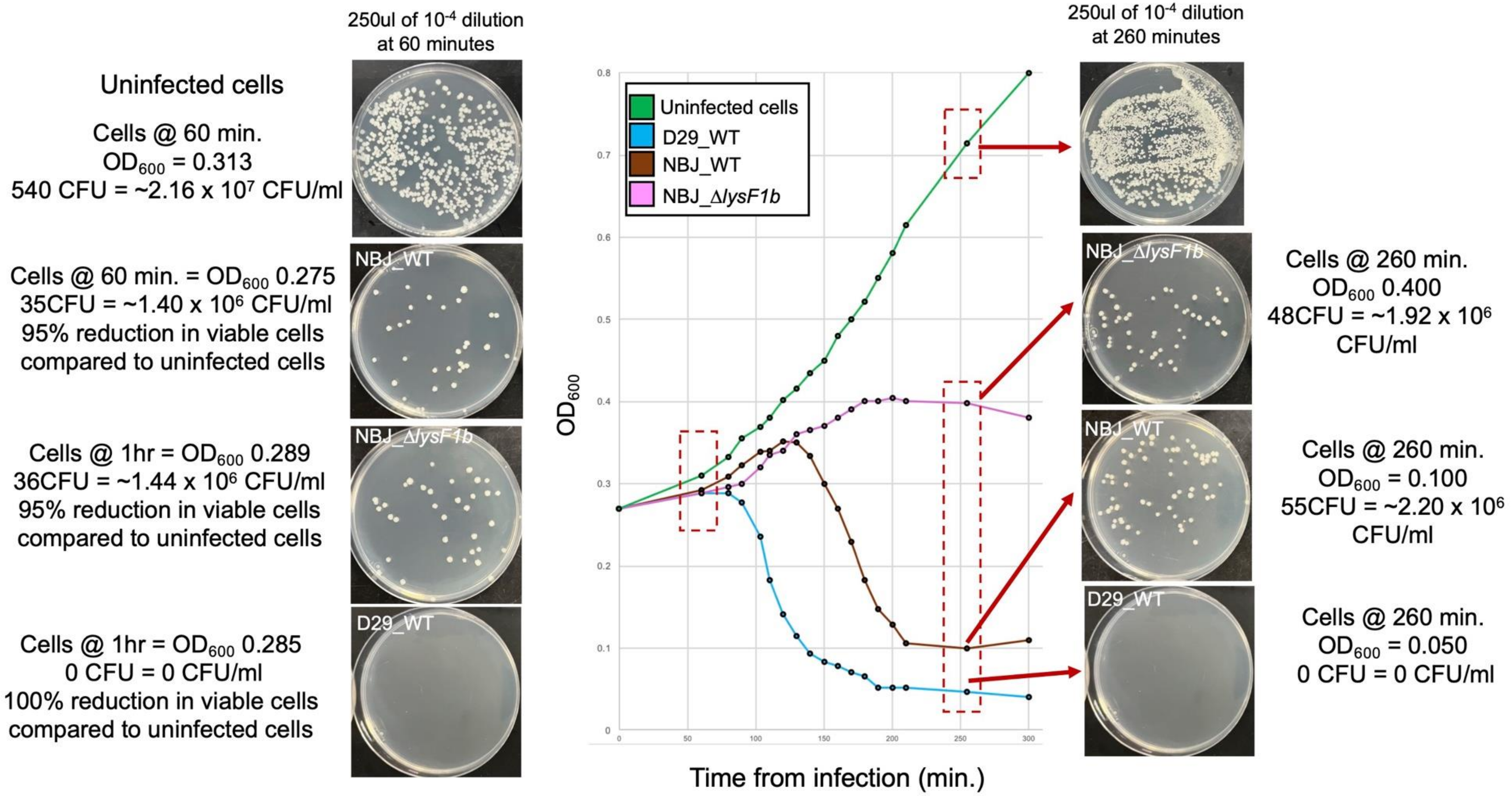

S11 Figure 10. Cell viability of *M. smegmatis* infected with NBJ phages. At T_0 a log growth culture of *M. smegmatis* at an OD_{600} of ~0.25 was infected with the indicated phage at an MOI of 10:1 and incubated at 37°C for 30 minutes without shaking. Control cultures (no phage) did not receive phage. At T_{30} minutes the culture was constantly shaken a 225rpm, aliquots removed at the indicated times and bacterial growth evaluated at OD_{600}. To assess the number of viable cells in the culture, aliquots were removed at T_{30} minutes and T_{260} minutes, diluted in 7H9 and 250ul spread on 7H10 agar and propagated at 37°C for 4 days. Phage D29 is a lytic phage and was used as a positive control.

S13 Figure 12. PCR analysis of Girr LRM variants and plaques

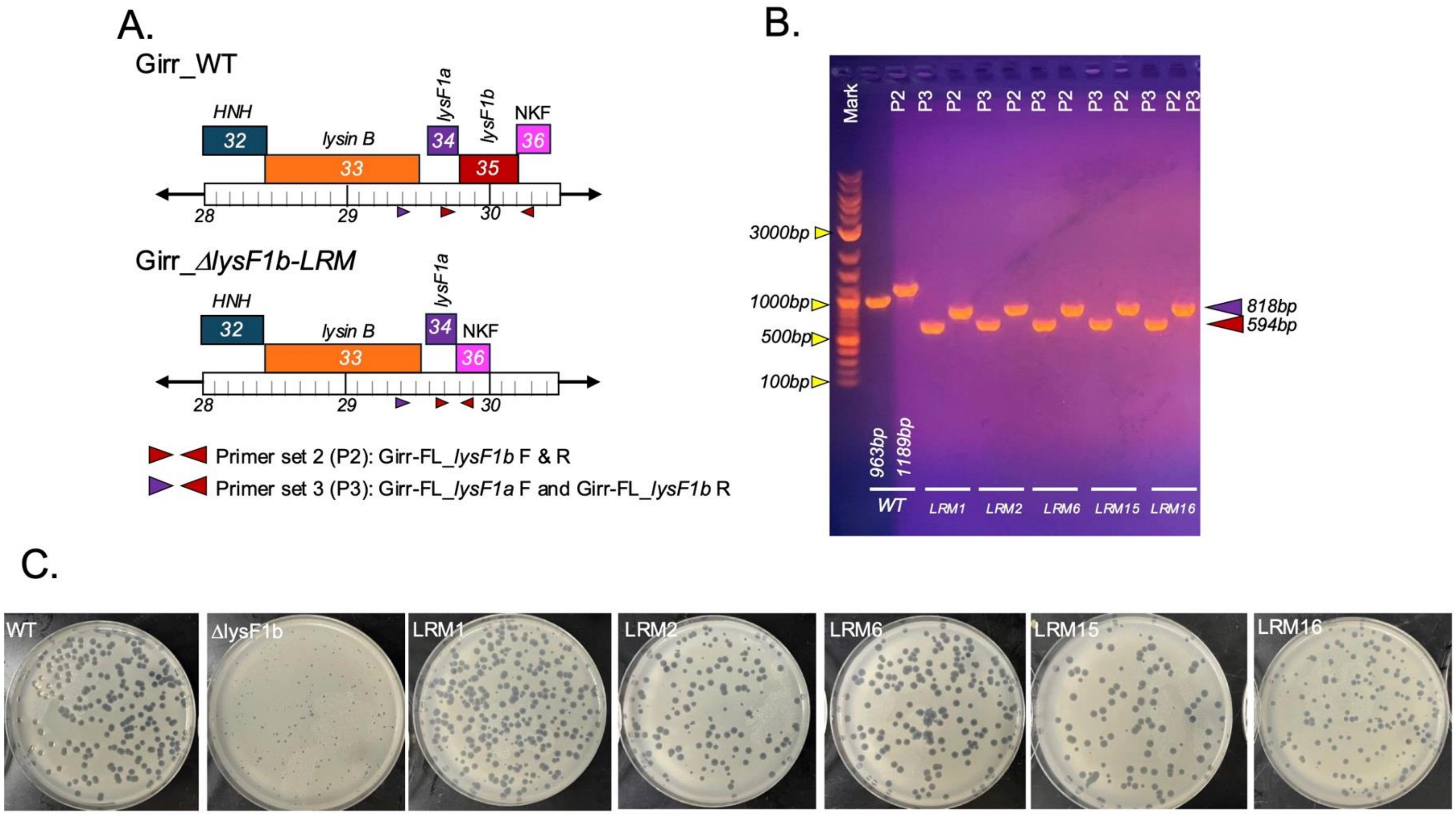

S13 Figure 12. PCR analysis of Girr LRM variants and plaques. A. Genomic structure of Girr_WT and Girr_Δ*lysF1b-LRM* showing location of PCR primer sets used to validate gene deletions. Primer set 2 (P2) uses primers Girr-FL_*lysF1b* (F and R) and generates a 933bp fragment in Girr_WT and 594bp with *lysF1b* deletion. Primer set 3 (P3) uses the forward Girr-FL_*lysF1a* and reverse Girr-FL_*lysF1b* and generates a 1189bp fragment in Girr_WT and or 818bp with *lysF1b* deletion. **B**. PCR of the indicated Girr LRM mutants. WT phage amplify with a 936bp band with P2 and a 1189bp band with P3. All LRM mutants are verified for the *lysF1b* deletion and amplify an 818bp band with P2 and 594bp band with P3. **C.** Plaque assay for Girr_WT, Girr_Δ*lysF1b* and the selected Girr LRM mutants showing plaque sizes. All plates were incubated for 36hrs. At 37ºC.

S14 Figure 13. Schematic of Girr_Δ*lysF1a*-LRM amino acid sequences and membrane topology

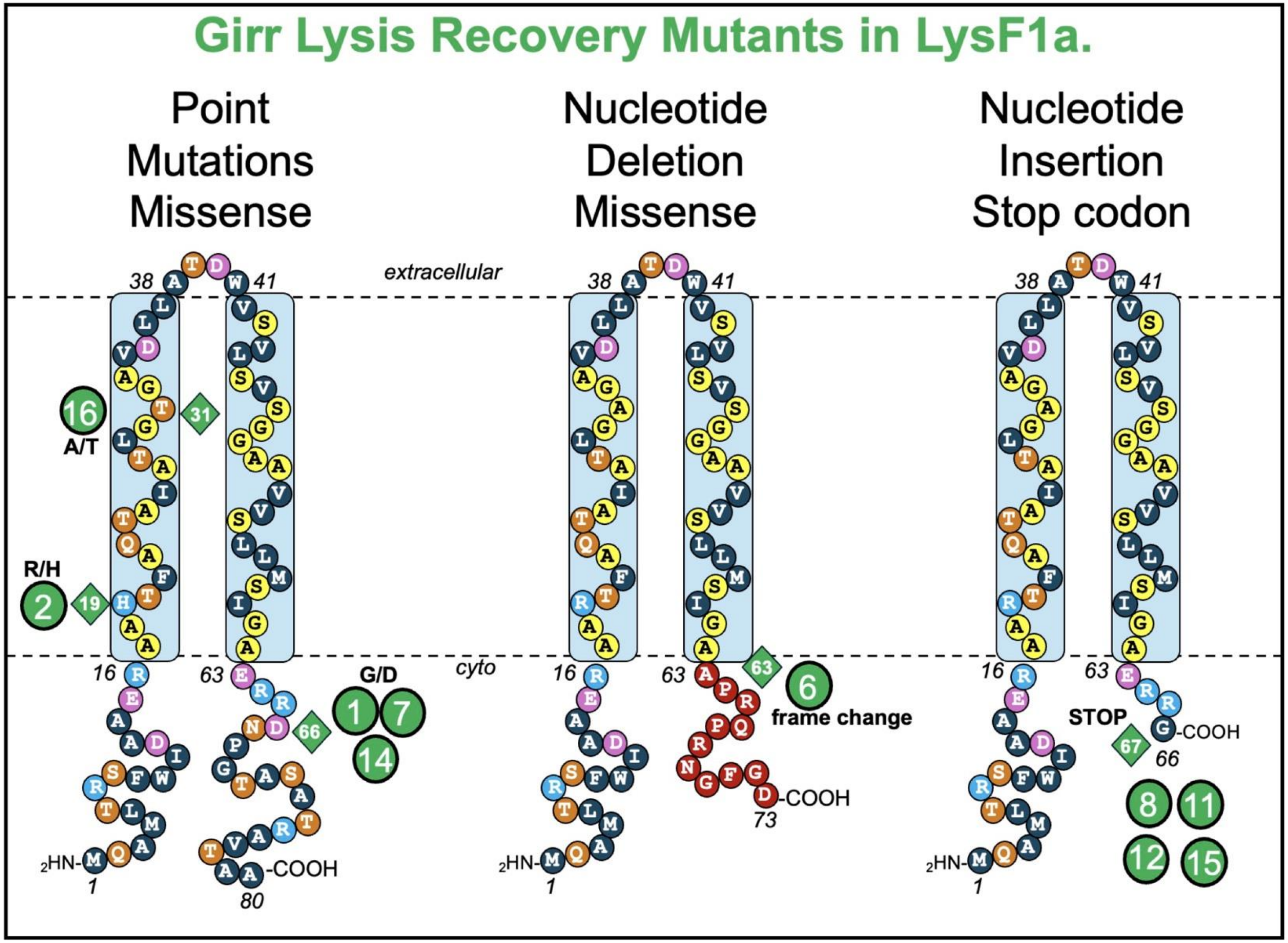

S14 Figure 13. Schematic of Girr_Δ*lysF1a*-LRM amino acid sequences and membrane topology. The impact of the nucleotide substitution, deletion and insertion mutations to the amino acids sequence of Girr LysF1a protein in the various LRMs. Green circles specify the Girr_Δ*lysF1a*-LRM clone. Green diamonds indicate the amino acid that was changed in each LRM. All amino acids are specified by the one letter code. Colors indicate the classification of the amino acid. Dark Blue: non-polar; Brown: polar; Pink: acidic; Light Blue: basic; Yellow: AG residues in the TMD helices predicted to be involved in homotypic and/or heterotypic helix interactions in pinholins [76, 77]. Red: missense translation and premature termination in Girr_Δ*lysF1a*-LRM6.

S15 Figure 14. AlphaFold3 analysis of Ms6 chaperone and Lysin A interactions

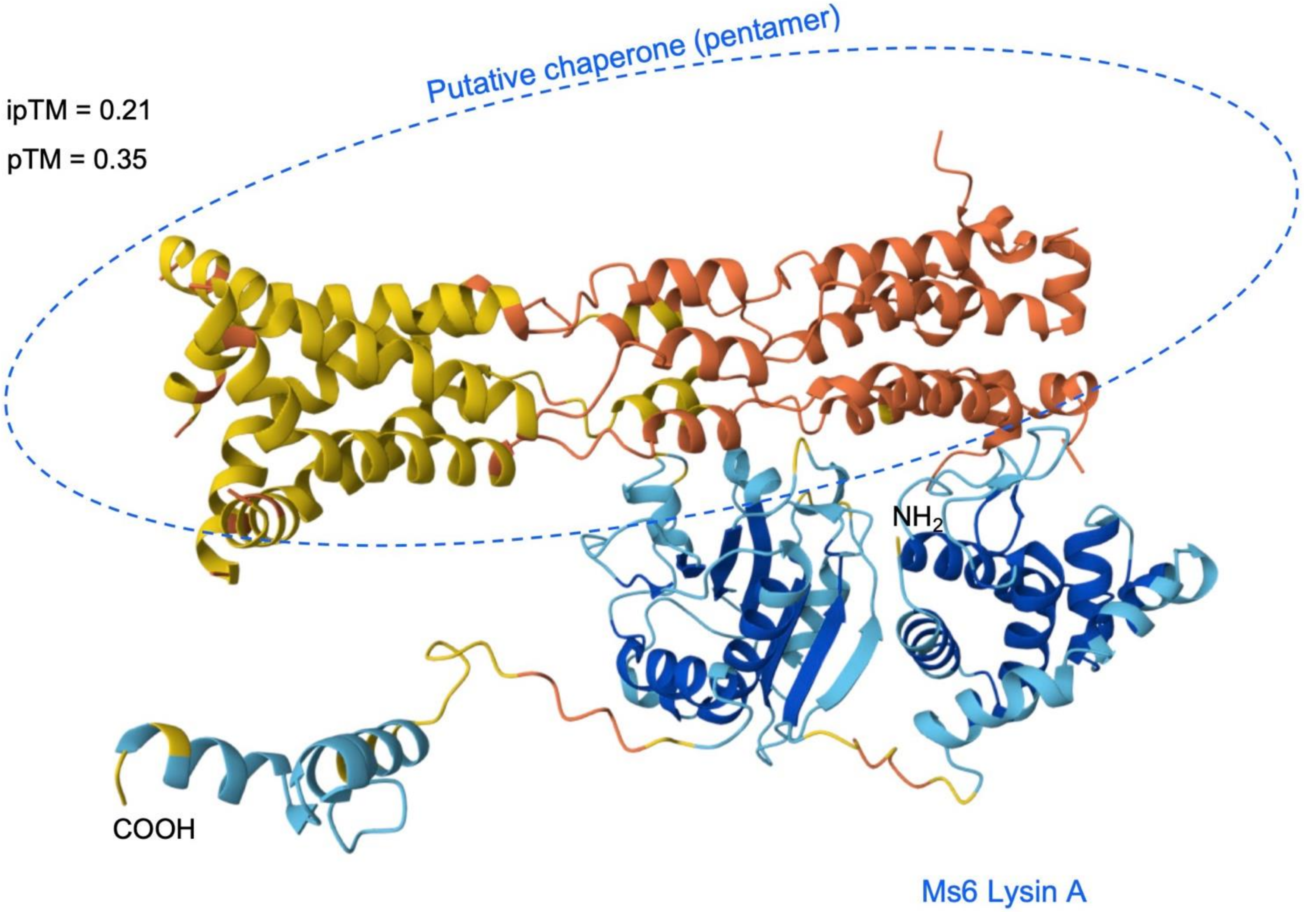

S15 Figure 14. AlphaFold3 analysis of Ms6 chaperone and Lysin A interactions. All protein sequences were evaluated using AlphaFold3 as described in Methods. The report on the interaction of these proteins proposes that the chaperone protein functions as a pentamer to bind with Ms6 Lysin A in the N-terminal region [50, 52]**.** Note the poor ipTM and pTM values for the predictions and lack of interaction of the chaperone to the N-terminal region of the Lysin A as proposed [50, 52]. Modeling with 1:1, 2:1, 3:1, and 4:1 ratio of chaperone and Lysin A did not improve binding interactions or the ipTM or pTM values.

S16 Figure 15. AlphaFold3 analysis of Girr gp29 and Lysin A interactions

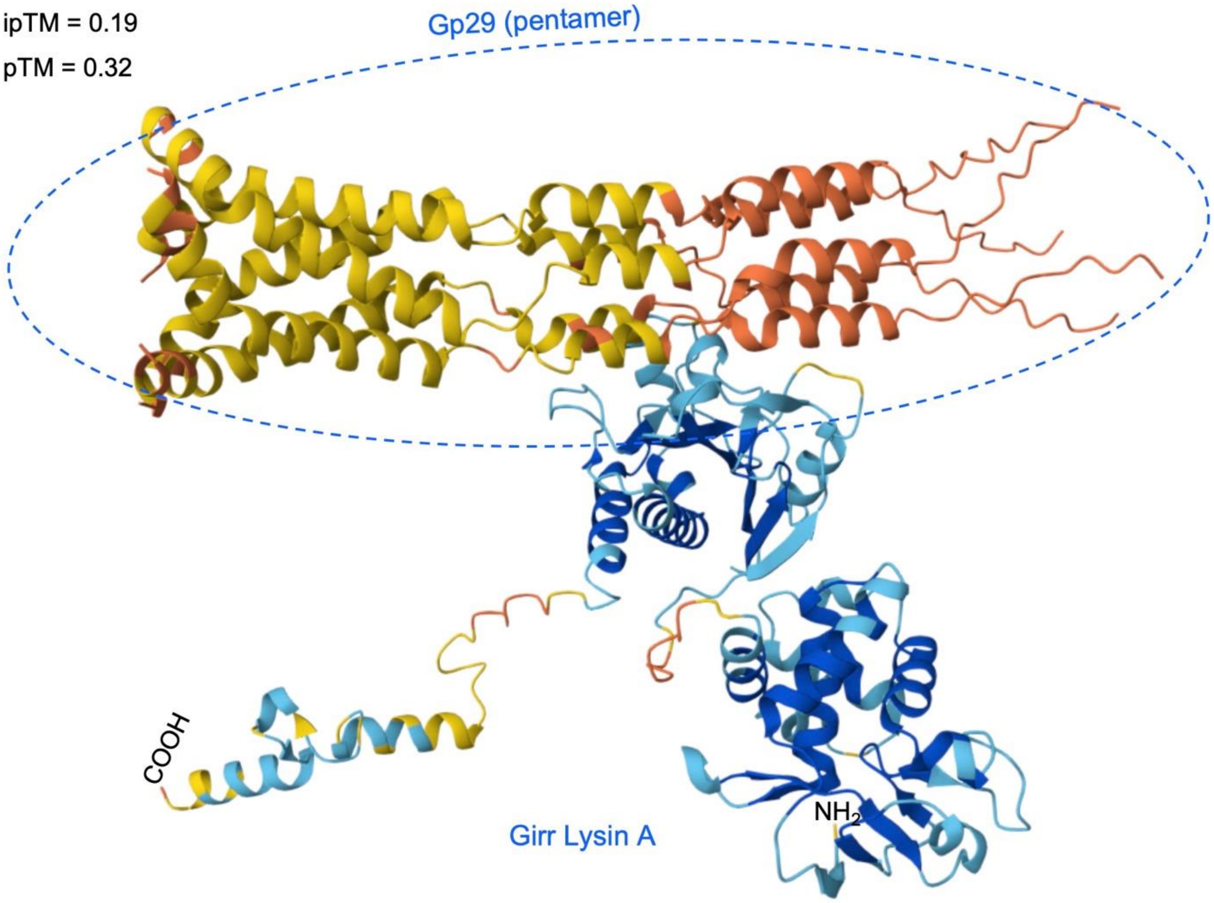

S16 Figure 15. **AlphaFold3 analysis of Girr gp29 and Lysin A interactions**. All protein sequences were evaluated using AlphaFold3 as described in Methods. The report on the interaction of these proteins proposes that the chaperone protein functions as a pentamer to bind with Ms6 Lysin A in the N-terminal region [50, 52]**.** Note the poor ipTM and pTM values for the predictions and lack of interaction of the chaperone to the N-terminal region of the Lysin A as proposed [50, 52]. Modeling with 1:1, 2:1, 3:1, and 4:1 ratio of chaperone and Lysin A did not improve binding interactions or the ipTM or pTM values.

S17 Figure 16. AlphaFold3 analysis of NBJ gp29 and Lysin A interactions

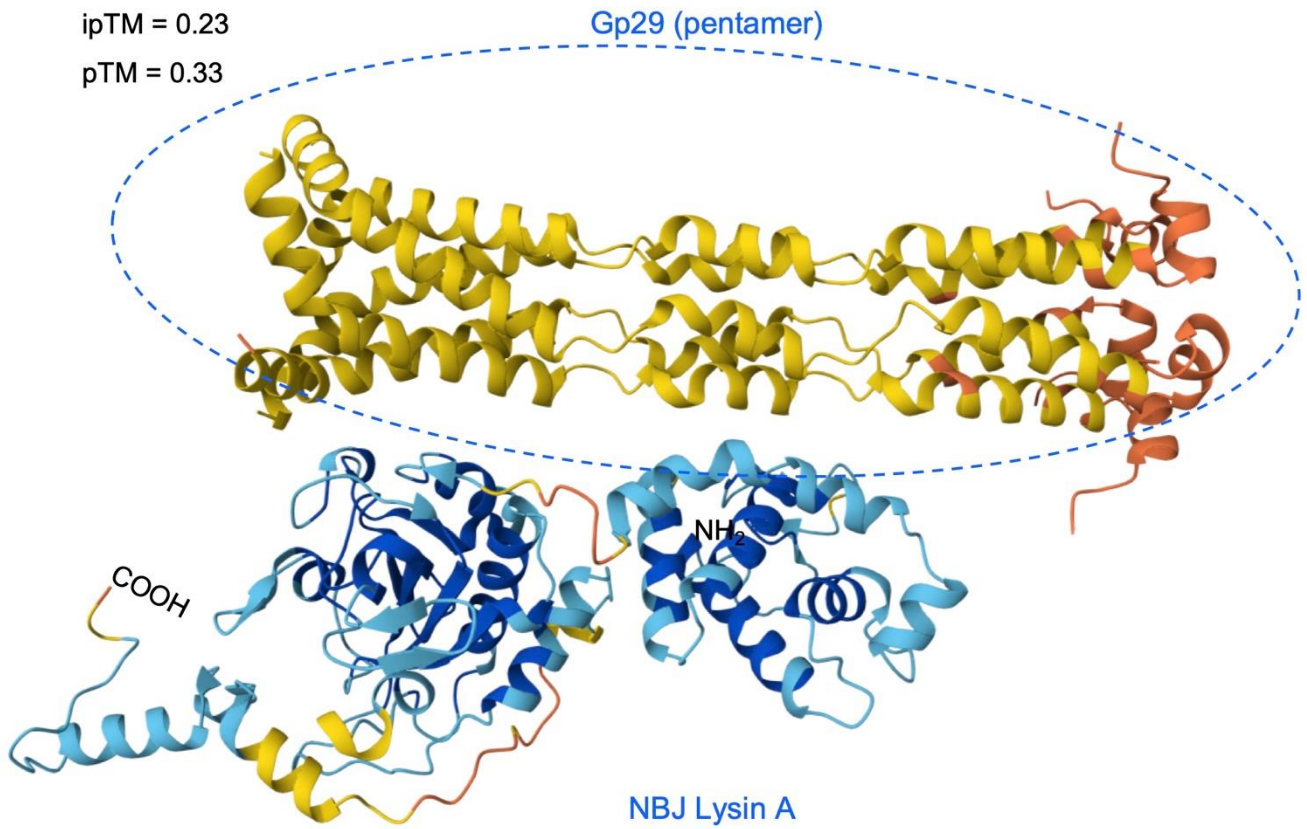

S17 Figure 16. AlphaFold3 analysis of NBJ gp29 and Lysin A interactions. All protein sequences were evaluated using AlphaFold3 as described in Methods. The report on the interaction of these proteins proposes that the chaperone protein functions as a pentamer to bind with Ms6 Lysin A in the N-terminal region [50, 52]**.** Note the poor ipTM and pTM values for the predictions and lack of interaction of the chaperone to the N-terminal region of the Lysin A as proposed [50, 52]. Modeling with 1:1, 2:1, 3:1, and 4:1 ratio of chaperone and Lysin A did not improve binding interactions or the ipTM or pTM values.

S18 Figure 17. Part I: Identification of genes that encode *lysF1a* and *LysF1b* homologs in phages that infect *M. smegmatis*

Cluster	Ave Genome Size (bp)	Number of phages																				
A1	51,632	243	5'		A	B		TM	mt	mt	2	1	mt									3'
A3	50,184	133	5'		A	B		TM	mt	mt	2	1	mt									3'
A5	50738	42	5'		A	B		TM	mt	mt	2	1	mt									3'
A8	52,052	12	5'		A	B		TM	mt	mt	2	1	mt									3'
A10	49,824	21	5'		A	B		TM	mt	mt	2	4	1	mt								3'
A19	50,283	1	5'		A	B		TM	mt	mt	2	1	mt									3'
AA	140,785	2	5'					TM	mt	mt	mt				A	2	1		4	B		3'
AI	101,258	2	5'	2	4	5	1	TM	mt	mt	mt				A	B						3'
D	64,803	23	5'					TM	mt	mt	mt				A	2		4	1	B		3'
E	75,466	144	5'					TM	mt	mt	mt				A	2	B	1				3'
F1	57,405	251	5'					TM	mt	mt	mt				A	B	2	1				3'
F2	56,209	8	5'					TM	mt	mt	mt				A	B	2	1		4		3'
F3	60,285	1	5'					TM	mt	mt	mt				A	B	2	1				3'
F4	57,081	4	5'					TM	mt	mt	mt				A	B	2	1		4		3'
F5	56,391	1	5'					TM	mt	mt	mt				A	B	2	1				3'
F6	59,328	1	5'					TM	mt	mt	mt				A	B	2	1		4		3'
F7	58,415	1	5'					TM	mt	mt	mt				A	B	2	1				3'
I1	48,048	6	5'					TM	mt	mt	mt				A	2	1	B				3'
I2	56,006	3	5'					TM	mt	mt	mt				A	2	4	1	B			3'
J	110,898	51	5'					TM	mt	mt	mt				A	B	2	1				3'
K7	60,430	1	5'					TM	mt	mt	mt				A	2	B		4	1		3'

TM	tape measure protein
mt	minor tail protein
A	lysin A
B	lysin B
1	1 TMD
2	2TMD
4	4TMD
5	5TMD
	Unknown function

S18 Figure 17. Part I: Identification of genes that encode *lysF1a* and *LysF1b* homologs in phages that infect *M. smegmatis*. Phages in the clusters listed in the table were analyzed for the presence of genes for *tape measure protein, minor tails, lysin A, lysin B* and associated genes encoding TMD proteins. All TMD proteins were evaluated for TMD domains and topology as described in Methods. The schematic is not to scale for size and simply shows the relative location of the annotated lysis genes in relation to the *tape measure protein* and *minor tails.* The order of the genes in relation to the lysis A are exact, with genes of unknown function shown as black boxes. All proteins predicted to have 1TMD or 2TMD have the same topologies as the LysF1a and LysF1b proteins but may not be grouped to the same phams and as shown in S21 Figure 20, may be <10% identical at the amino acid level. The intent of the schematic is to show that lysis cassettes containing endolysins and genes encoding a 1TMD and 2TMD proteins are a common feature across the >2,600 annotated phages that infect *M. smegmatis*. Phages in clusters A1, A3, A5, A8, A10, A19 and AI have the TMD genes in a separate cassette in relation to the genomic location of the lysins.

S19 Figure 18. Part II: Identification of genes that encode *lysF1a* and *LysF1b* homologs in phages that infect *M. smegmatis*

Cluster	Ave Genome Size (bp)	Number of phages																
L1	72,995	20	5'	TM	mt	mt	mt				A	B	2	1				3'
L2	74,987	41	5'	TM	mt	mt	mt				A	B	2	1				3'
L3	74,117	24	5'	TM	mt	mt	mt				A	2	B	■	1			3'
L4	76,614	12	5'	TM	mt	mt	mt				A	B	2	1				3'
L5	74,943	2	5'	TM	mt	mt	mt				A	B	2	1				3'
M1	81,175	18	5'	TM	mt	mt	1	mt	A	■	2	B						3'
M2	82,578	6	5'	TM	mt	mt	1	mt	A	■	2	B						3'
M3	82,676	1	5'	TM	mt	mt	1	mt	A	■	2	B						3'
N	42,913	49	5'	TM	mt	mt	mt				A	2	1					3'
O	71,102	34	5'	TM	mt	mt	mt				A	B	2	2	2	1		3'
P1	47,555	48	5'	TM	mt	mt	mt				A	B	2	1				3'
P2	49,658	1	5'	TM	mt	mt	mt				A	B	2	1				3'
P3	49,808	1	5'	TM	mt	mt	mt				A	B	2	1				3'
P4	48,927	2	5'	TM	mt	mt	mt				A	B	2	4	1			3'
P5	49,809	2	5'	TM	mt	mt	mt				A	B	2	4	1			3'
P6	50,513	1	5'	TM	mt	mt	mt				A	2	1					3'
R	71,339	10	5'	TM	mt	mt	mt				A	2	4	1	B			3'
S	64,976	20	5'	TM	mt	mt	mt				A	B	2	4	1			3'
Singletons	various	6	5'	TM	mt	mt	mt				A	B	2	1	■	4		3'
T	42,724	8	5'	TM	mt	mt	mt				A	2	1					3'
U	67,818	5	5'	TM	mt	mt	mt				A	B	2	4	1			3'
V	77,907	5	5'	TM	mt	mt	mt			4	1	A	2	B				3'
X	88,037	2	5'	TM	mt	mt	mt				A	B	2	1				3'
Y	76,507	6	5'	TM	mt	mt	mt			2	A	A	B	4	1			3'
Z	50,807	2	5'	TM	mt	mt	mt				A	B	2	1	■	4		3'

TM	tape measure protein
mt	minor tail protein
A	lysin A
B	lysin B
1	1 TMD
2	2TMD
4	4TMD
5	5TMD
■	Unknown function

S19 Figure 18. Part II: Identification of genes that encode *lysF1a* and *LysF1b* homologs in phages that infect *M. smegmatis*. Phages in the clusters listed in the table were analyzed for the presence of genes for *tape measure protein, minor tails, lysin A, lysin B* and associated genes encoding TMD proteins. All TMD proteins were evaluated for TMD domains and topology as described in Methods. The schematic is not to scale for size and simply shows the relative location of the annotated lysis genes in relation to the *tape measure protein* and *minor tails.* The order of the genes in relation to the lysis A are exact, with genes of unknown function shown as black boxes. All proteins predicted to have 1TMD or 2TMD have the same topologies as the LysF1a and LysF1b proteins but may not be grouped to the same phams and as shown in S21 Figure 20, may be <10% identical at the amino acid level. The intent of the schematic is to show that lysis cassettes containing endolysins and genes encoding a 1TMD and 2TMD proteins are a common feature across the >2,600 annotated phages that infect *M. smegmatis*.

S20 Figure 19. Identification of LysF1b homologs in phages that infect *M. smegmatis*

>Rem711_gp38_Z
MTVWNPALWDDFGVVGIVVAVGTLFVVAQLRGWIVLGRQHREIVAMKDFTIAEQQAREAKDAETIATLSQAVTEKTATTRILAAVPDTTGSAP
>Avani_gp38_F2
MTMWSADFWNGLGVVGVVLIVGFLFLLSLQRGWLVLGIHHREVVEGLRRENAAYVQRAKQDAEAIAEFGKADAKRAFNEDGVAKILAALRETITTGGGQ
>Bongo_gp33_M1
MIDVGMIATAIGALATGGVLTTVWGSFFAPKRDKTSEAEAIQKLGAEIRQEIREENAALRERMDVVVNAVISLTDLLDDLFPKITGLSDEERIALRHKINLAKRAT
>Hilltopfarm_35_Y
MVGSDVVATMVALATGILGGGAGAAFWAGFFKRNTDRADAANAISQGAVGLLTRADKEIAKLEKSNGELKAVAGPLLDAAEQMLPLLPTGDPRAAAWAKAIHDARLVL
>Philly_gp19_B3
MSVSLVFTLLGSAGFLAGAVALFQFLNTRKATKSKGSADALLAWRQFTSTAVGDAVDDRDRMKVERDSLHLIRASLIDLVQDLIVALRRHGATPEDVEPYQDRLDEVRAL
>Mitti_gp34_K4
MSTEVIVALCSLVGVVVTAILAHRAGGRAALRGAETADWKAFTDSLSDRLLRVEERLAKAEERTAKAEDRANRAESLYRIAITYLRQVVQWCGERHGEKLPEVPAELAGDL
>Clarkson_gp61_S
MAVAEIISTLVTGGIGAAIGSIAVAFINAWSRKGESRASAADLVTNAGVGLIDRLERTNERLDKENKHLRQALLTLTDVIDDMLPSLPADDDATERMRTKLQKANREAKMVV
>Megsy_gp33_K1
MSDGITAFEAVAALAGSSVLSVSVGALLARRRDNFKALTDVLMDRVTKLETRVDTVETKLDAEQIAHEKTRMVLAAREMTLAAARAFIRTVQRWAAGDRVDPIPTPPDEVMAE
>Abrogate_31_A1
MTEVLDWLAVASGPAGIAIGIYGEKWRSRRREPAEIEKTEAEASQIFVETAVTLIAPLKAEIADLTVRVNQLEEENYTTKTRLQLSIDYIRVLQTWISKHIPGRKPPAPPAELLL
>Cosmo_gp48_V
MDWWQTTLLSVLTAGVGIMLTMLLNRNKDANALAHKAINSTRAQFEVIETLKTISWLQAVRLDEWENWGREATQVFNETARIHEECGCNTSHLPRMPSPPEGHLDLRRYWRQIEEAVADKE
>Girr_gp35_F1
MIWESVREAVDAAYQPDDGIDLIGLLIIGLPSTIAAIGTGIVGVLTVRGQRKGRERARRIDAKTYEIHEQTVNTHDTNMRDDLDEIRDLVRDGFKQIQRDIGGLREELRTERLERIEGDKRRDR
>Che9c_gp29_I2
MNAWGPAILSSGIAGAAVSLILAIMNRRHTNALARQVENAAEREEVERDNLLAEATSKWSKLLDETRTEAYKEIDRRCRRCEAALALRDESVDRLIDAVTALIPLVPAGAAELAAVHTAIQAARRARYADED
>Aminay_gp34_K7
MNTQLIVNLLLASGTLATALLGVMSYRQNRRKGDADIEHSQATTTEVVNRAAQINETREMEREKFWRSTLADTERRLEGELSNVRAELANLKNGINKHVSWDFKAIRELRLLGSDIEDPPSLLYVPDDDERK
>Candle_gp38_R
MKVLESLITQAALVTVAAISAVGGIYSTYANRKQNSATADKTKKEGEQVGDQTWIRRLEALSQDFEKLQKLSDERFERLVEIEMLITEHVSWDFRIMRLLREHGIEAETPPSLVYVRHKLKEAQEQEKSLGETG
>Bruin_gp36_E
MTGEWITVVAGQWPWLILVAVFLMLIPQAVKAIETVKGLLAPLFERFTPAAKRKDKRDDINAKVADLEAQVTFLTEQVANLRARDRMYWAWVISDQEWHRKVELEAAERGWELPHHVSFDEFYDEWIIQHPIPRPYQL
>CiCi_30_A4
MSVELFQHLPQNWVGLFAVIMFVVYIAAQVVEKFEKIAKFLPGGTWWHEKQKNKRNRRSEWVTEDNEVIQALQNQVSSIAGQLATVQETVRVFSAWSVYDARWHHKTSVVNANGTCLLPDHLDYFAFERLWRVDPMEASKLPA
>Catdawg_gp72_O
MSVLAEAATQPTGITPAVATIIVAVVTVLGGVVGAIVSYWLNRNNNSADTAEKISRAWVPVFTQYDTALEQVQKQCDACRKELNEAAERWRVAEERATDYERRLTAAEQRERTQTEVLRTLVRVYDANDPTEIEAAITQIRQMLRG
>Chill_gp40_D1
MNGNVDVGWILTFLAIVSASGPVLSVVLDYRSRKRQAEAEANNKDADTGIKKVSEQELSQRVLSMNDERWITREKRWQEREDALEREIQELRKEMDNLRSDMNAYLEFIANDEQWHFYDRMHRIQNDLPAPQDRITFQQFMESRREAAGRM
>Fowlmouth_gp33_AB
MNNWLQGIGIIVAAIVSLVGLFISIRNRKRDTKAVEAKTNLDETQQASIVKQLADSTEVMYLKRLESFKDDIRLMRAELDAARKDSESSRQEVALARDEARKAREEASLFEDFFFNHHSRWDRTLLMIARENGWEIPDPPSWLIFLRSKGMNDDD
>Damien_gp37_H1
MFADLDKVNEVLGQNVLVIVFIVAVVWFIVTKLAETKDSFAKILGPIGRRILHAKEKREERHREEVRLEAKRVLKEAGTLEPPDYQTVKRRLSNVLEEVSAQAATIREVQLENRGLRTYIMQDEDWHWEDERSAIRENRTVRQRVSFDEFMDDFMHRNL
QRERGLGNGTPI

S20 Figure 19. Identification of LysF1b homologs in phages that infect *M. smegmatis*. Phages in all clusters in the Actinobacteriophage database that infect *M. smegmatis* were evaluated to identify gene/s that encode a protein with a single TMD. Within the PhagesDB.org database, the identified proteins were grouped into 20 different phams as of 12/2/2025. A representative protein from each pham is presented and the phage name, gene product number and cluster are identified. All proteins were predicted to have an N-out-C-in topology and the proteins are highlighted as described in Figure 2. The TMD is identified with green font. The basic KR amino acids directly after the TMD are highlighted in yellow with red font. Acidic DE residues are highlighted in blue. Basic KRH residues are highlighted in blue. The proteins ranged in size from 93 amino acids (Rem711: gp38), to 171 amino acids (Damien: gp37).

S21 Figure 20. Percent identity of LysF1b homologs

	Philly	Cosmo	Megsy	Mitti	Catdawg	CiCi	Bruin	Girr	Bongo	Damien	Abrogate	Chill	Aminay	Candle	Avani	Rem711	Fowlmouth	Che9c	Clarkson	Hilltopfarm
Philly	100																			
Cosmo	24.53	100																		
Megsy	12.51	10.94	100																	
Mitti	17.72	17.91	29.21	100																
Catdawg	14.77	25.01	18.75	25.61	100															
CiCi	7.06	4.08	8.64	6.41	14.14	100														
Bruin	6.91	4.44	12.01	12.82	7.37	24.44	100													
Girr	12.01	14.55	12.21	8.16	20.29	10.39	14.47	100												
Bongo	6.91	3.67	1.85	1.89	5.71	11.39	18.92	18.03	100											
Damien	9.52	12.91	10.01	10.34	3.57	15.51	16.67	13.95	15.22	100										
Abrogate	9.86	12.28	15.38	12.91	10.13	9.21	16.25	9.52	15.05	17.17	100									
Chill	9.46	4.35	14.92	13.75	8.74	13.51	21.15	14.71	25.51	16.31	16.67	100								
Aminay	11.43	12.07	15.12	14.67	12.12	11.88	21.21	23.33	13.95	13.01	22.01	23.08	100							
Candle	16.44	4.17	8.01	13.24	13.98	10.01	8.74	13.85	15.91	14.96	18.18	16.67	27.12	100						
Avani	8.57	9.46	13.89	21.62	20.01	12.51	9.31	21.43	16.13	12.51	20.64	14.29	9.62	10.01	100					
Rem711	9.09	13.24	15.62	21.62	10.87	4.26	9.31	21.82	12.07	21.01	15.25	16.95	4.17	10.87	38.71	100				
Fowlmouth	12.77	8.43	10.98	8.33	12.62	10.58	19.23	19.28	8.43	11.41	16.31	13.51	20.39	18.37	14.71	12.12	100			
Che9c	14.29	19.19	14.06	13.24	14.29	4.62	6.67	14.86	14.29	12.05	11.39	12.09	8.64	5.63	18.89	19.05	20.95	100		
Clarkson	3.71	10.81	14.04	12.01	16.22	7.46	12.91	21.21	13.71	8.75	10.96	13.1	16.01	8.23	8.57	15.62	22.51	20.39	100	
Hilltopfarm	22.01	16.22	16.98	17.39	17.14	9.52	15.52	11.29	14.49	18.42	10.14	12.5	9.86	10.29	10.01	10.94	19.74	25.25	26.85	100

S21 Figure 20. Percent identity of LysF1b homologs. All proteins shown in S20 Figure 19 were evaluated by the multiple sequence alignment tool in Clustal Omega. The precent amino identity across all proteins is shown. Girr LysF1b is highlighted in yellow.
