# Supplementary material for: Genetic analysis of F1 cluster phages that infect Mycobacterium smegmatis identifies two distinct holin-like proteins that regulate the host lysis event
Source: PLoS One. 2026 Apr 7;21(4):e0339202. doi: 10.1371/journal.pone.0339202 (PMC13056207; doi:10.1371/journal.pone.0339202)
Supplement: Attachment — Submitted filename: Comments to Author_PLOS ONE 2026.docx [file pone.0339202.s003.docx]

**Comments to Author:**

In the current study, authors have investigated the genomic and functional comparison of Two Distinct Holin-Like Proteins (LysF1a and LysF1b)from Phages Girr and NormanBulbieJr (NBJ) of *Mycobacterium smegmatis.* They have performed the recombineering for the deletion of both the genes and investigated the plaque morphology in mutant phages. They have also analysed the lysis recovery mutants and did the functional genomics for the same. Authors have done good phage biology experiments, and presented in better way. Further, there are some concern which need to be addressed before final publication.

1. Line 125-155, In introduction section, authors can narrow down the result description, which is already being done in result and conclusion. It apears as repeatation of same information.
2. Advised to merge Fig1 and 2 together.
3. Figure 3 can be moved to supplementary
4. Marker lane in Fig 5B is smeared; it is advised to use another figure with improved marker lane.
5. Figure 9, In graph A, B, curve for no phage seems truncated, and not upto end of kinetics. It is advised to give full curve for better comparison.
6. Figure 10; What is impact of KCN or CHCL_3_ on wild type phage treated bacterial cell growth.
7. The measurement of CFU count but not absorbance is ideal for viability counts of bacterial cells. Although one can calculate the growth rate from growth kinetics data by using the formula of growth rate (refer this article PMCID: PMC12572221), and can perform the statistical analysis among different group for the statistical significance.
8. It is advised to merge some figure to reduce the number of figure in final manuscript.
